# Comparable clinical and functional outcomes between biological and synthetic grafts for medial patellofemoral ligament reconstruction: A systematic review and meta‐analysis

**DOI:** 10.1002/jeo2.70627

**Published:** 2026-02-19

**Authors:** Saran Singh Gill, Pratik Ramkumar, Akash Patel, Fahad Siddique Hossain, Raj R. Thakrar

**Affiliations:** ^1^ Faculty of Medicine Imperial College London London UK; ^2^ Royal Free London NHS Foundation Trust London UK; ^3^ Faculty of Medical Sciences University College London London UK; ^4^ Walsall Healthcare NHS Trust Watford UK; ^5^ The Lister Hospital NHS Trust Stevenage UK

**Keywords:** allograft, autograft, medial patello‐femoral ligament reconstruction, MPFL, synthetic graft

## Abstract

**Purpose:**

The medial patellofemoral ligament (MPFL) is an essential static stabiliser of the patella. In patients with patellofemoral dislocations and MPFL damage, surgical intervention is often necessary to restore stability. This typically involves reconstruction using either an autograft, allograft or synthetic graft. This study aimed to evaluate long‐term outcomes of biological grafts (autografts and allografts) versus synthetic grafts, by analysing parameters such as stability and complication rates.

**Methods:**

A systematic search on Ovid, Medline, Embase, PubMed and Cochrane electronic databases was performed. Studies were included if they enroled adult patients (≥18 years) who had undergone isolated MPFL reconstruction for chronic patellofemoral instability, with a minimum follow‐up of 12 months. Data were pooled through meta‐analysis using an inverse‐variance and mixed‐effects model in RStudio to generate standardised mean differences or rate ratios with 95% confidence intervals.

**Results:**

Thirty‐two studies were included in this study, with 1508 patients. 224 were treated with synthetic grafts and 1284 with biological grafts. No statistically significant differences were observed between synthetic and biological grafts across all primary and secondary outcomes: Kujala score (*Q* = 1.24, *p* = 0.27), Lysholm score (*Q* = 1.62, *p* = 0.20), Tegner score (*Q* = 0.11, *p* = 0.74), postoperative complication rate (*χ*² = 0.0.1, *p* = 0.94), nor redislocation rates (*χ*² = 0.34, *p* = 0.56). These findings indicate comparable functional outcomes and safety profiles for biological versus synthetic grafts.

**Conclusion:**

The findings of this study have shown clinical and functional outcomes to be similar when comparing synthetic and biologic grafts for MPFL reconstruction. These findings support the use of synthetic grafts as a viable alternative in the surgical treatment of chronic patella instability and emphasise the need for a meticulous and informed approach to graft selection for MPFL surgery.

**Level of Evidence:**

Level IV.

**Abbreviations**
BMI: body mass index
CDI: caton–deschamps index
CI: confidence interval
df: degrees of freedom
IKDC: International Knee Documentation Committee
*I*²: I‐squared statistic
KOOS: knee injury and osteoarthritis outcome score
LARS: ligament augmentation and reconstruction system
MPFC: medial patellofemoral complex
MPFL: medial patellofemoral ligament
MPFL‐R: medial patellofemoral ligament reconstruction
MRI: magnetic resonance imaging
OA: osteoarthritis
OR: odds ratio
PF: patellofemoral
PF OA: patellofemoral osteoarthritis
PRISMA: preferred reporting items for systematic reviews and meta‐analyses
PROM: patient‐reported outcome measure
Q angle: quadriceps angle
RCT: randomised controlled trial
RoB: risk of bias
ROBINS‐I: risk of bias in non‐randomised studies—of interventions
ROB 2: cochrane risk of bias 2.0 tool
RR: risk ratio
SD: standard deviation
TTO: tibial tubercle osteotomy
TT–TG: tibial tubercle–trochlear groove distance
VAS: visual analogue scale
τ²: Tau‐squared statistic (between‐study variance in meta‐analysis)
*χ*²: Chi‐squared statistic

## INTRODUCTION

The patellofemoral joint is vital as it facilitates flexion, extension and contributes to rotation of the knee joint. The medial patellofemoral ligament (MPFL) is important in providing stability to this joint, in its function as a primary stabiliser of the patella [58]. It serves as a restraint to maintain lateral patellar translation within the 0° to 30° flexion range, contributing to approximately 60% of lateral patellofemoral stability.

Patellofemoral instability can develop through either traumatic injury or non‐traumatic episodes, particularly in patients exhibiting anomalous anatomical features. Acute traumatic patellar dislocation carries a substantial risk of associated MPFL damage. In these cases, nonoperative therapies may be insufficient to restore patellar stability, as recurrent dislocation poses an ongoing challenge. Consequently, surgical intervention is important to consider to avoid further episodes of instability, patellofemoral osteoarthritis and potential loss of activity in affected individuals [4, 33, 58].

MPFL reconstruction is now regarded as the standard surgical approach for restoring patellofemoral stability, with primary repair or imbrication generally reserved for selected acute injuries rather than routine management of recurrent instability. MPFL reconstruction can be accomplished using autografts, allografts or synthetic grafts. Autografts, specifically those harvested from the hamstring tendons, are widely utilised owing to their favourable biomechanical attributes, geometric properties and hyphen‐availability [1, 27]. Both autograft and allograft‐based MPFL reconstruction have shown positive outcomes with measurable increases in Kujala score, which is recognised as a validated metric in the assessment of patellar stability [37, 39, 41]. However, despite the apparent advantages and positive clinical outcomes, several associated complications have been described, including postoperative pain, patellar fractures, donor‐site morbidity and increased infection rates [41]. As a result, synthetic woven grafts have emerged as an alternative [2, 10, 15, 48, 56].

Previous studies comparing biological and synthetic grafts for MPFL reconstruction have found comparable outcomes between them [28, 35, 36, 59]. These results may encourage the use of synthetic polyester tape (PT), which have been shown to yield similar clinical outcomes to autografts [11]. Despite these findings, synthetic materials have not yet been widely adopted as an alternative graft option. Although several reviews have been published on this topic, most included limited study numbers and small patient cohorts, did not directly compare graft types or did not perform a meta‐analysis [5, 10, 25, 35, 36, 56]. There also remains a scarcity of data evaluating long‐term outcomes, particularly for synthetic grafts.

Accordingly, this study aimed to provide a comprehensive and up‐to‐date analysis evaluating stability and redislocation rates for biological versus synthetic grafts in MPFL reconstruction, with the goal of informing optimal clinical decision‐making. We hypothesised that outcomes following MPFL reconstruction would be comparable between biological and synthetic grafts.

## METHODS

The study was registered on PROSPERO (International prospective register for systematic review, CRD42020220150). A systematic review and meta‐analysis were conducted according to the Cochrane handbook of systematic reviews and meta‐analyses and followed the preferred reporting items for systematic reviews and meta‐analyses (PRISMA) statement guidelines [19, 42].

### Literature search

The search was undertaken by two review authors (S.S.G. and P.R.) on 9th March 2025 encompassing MEDLINE, Embase, PubMed and Cochrane without any time limit. The full search strategy is detailed in the Supporting Information: Table S1.

### Selection criteria

The inclusion criteria comprised studies that enroled adult patients (≥18 years) with patellar instability who had undergone MPFL reconstruction using either a biological graft, defined as an allograft or autograft or a synthetic graft, with a minimum follow‐up of 12 months. The exclusion criteria included abstracts, letters to the editor, case series with fewer than five patients, case reports, commentaries, expert opinions, cadaveric studies, studies involving MPFL alongside additional procedures and research involving the paediatric population. Two review authors (S.S.G. and P.R.) independently assessed studies for eligibility. Any discrepancies were resolved by consensus. The titles and abstracts of the electronic database search were initially screened using covidence. Full text articles of the studies meeting the inclusion criteria were then reviewed. Biological grafts were analysed together given their broadly comparable clinical outcomes reported in previous literature [3, 18, 38].

### Methodological quality assessment of included studies

The quality of the included studies was assessed using the Cochrane Risk of Bias 2.0 tool for randomised controlled trials and the ROBINS‐I tool for non‐randomised studies [53, 54]. Each tool consists of a series of domains with signalling questions to evaluate specific sources of bias and confounding. Responses to each question were recorded as ‘low risk’, ‘some concerns’ or ‘high risk’ for the Cochrane tool and as ‘low risk’, ‘moderate risk’, ‘serious risk’ or ‘critical risk’ for ROBINS‐I. Following domain‐level assessment, each study was assigned an overall quality score of ‘good‘, ‘fair’ or ‘poor’.

### Data extraction

Two authors (S.S.G. and P.R.) independently assessed the included studies and extracted the following data:

1. Characteristics of studies and population, including study design, study sites, inclusion criteria, sample size, type and source of grafts, duration of follow‐up, postoperative complications and conclusions
2. Baseline characteristics of the enroled participants, such as the number of patients, age and proportion of female patients.
3. Outcomes, including Kujala score, Lysholm score, Tegner score, International Knee Documentation Committee (IKDC) score, complication rates, redislocation rates, and patients with a positive patellar apprehension test.

### Data synthesis and statistical analysis

Data were first summarised using descriptive statistics to summarise study characteristics, patient demographics and reported outcomes. When continuous endpoints were reported as medians with ranges or interquartile ranges, they were converted into approximate means and standard deviations using the Hozo and Wan methods for ranges and an interquartile range (IQR)‐to‐standard deviation (SD) conversion by dividing the interquartile range by 1.35 [23, 63]. Analyses were conducted in RStudio, with biological and synthetic graft groups evaluated separately. Postoperative Kujala, Lysholm, Tegner and IKDC scores were pooled using the metamean function under a DerSimonian–Laird random‐effects model to generate summary means and 95% confidence intervals. The rates of complications, patellar apprehension and redislocation were pooled using the metaprop function with Freeman–Tukey double‐arcsine transformation and inverse‐variance weighting to yield summary proportions and their 95% confidence intervals. Heterogeneity was assessed using Cochran′s *Q* statistic and the *I*² metric for continuous outcomes, while pooled event rates were evaluated using the chi‐squared statistic and *I*². A between‐groups *Q‐*test was performed to compare biological and synthetic graft subgroups and determine whether their pooled estimates differed significantly. Publication bias was assessed using funnel plot inspection and Egger′s regression test when ten or more studies were available.

## RESULTS

### Systematic review and characteristics of the included studies

**Figure 1:**
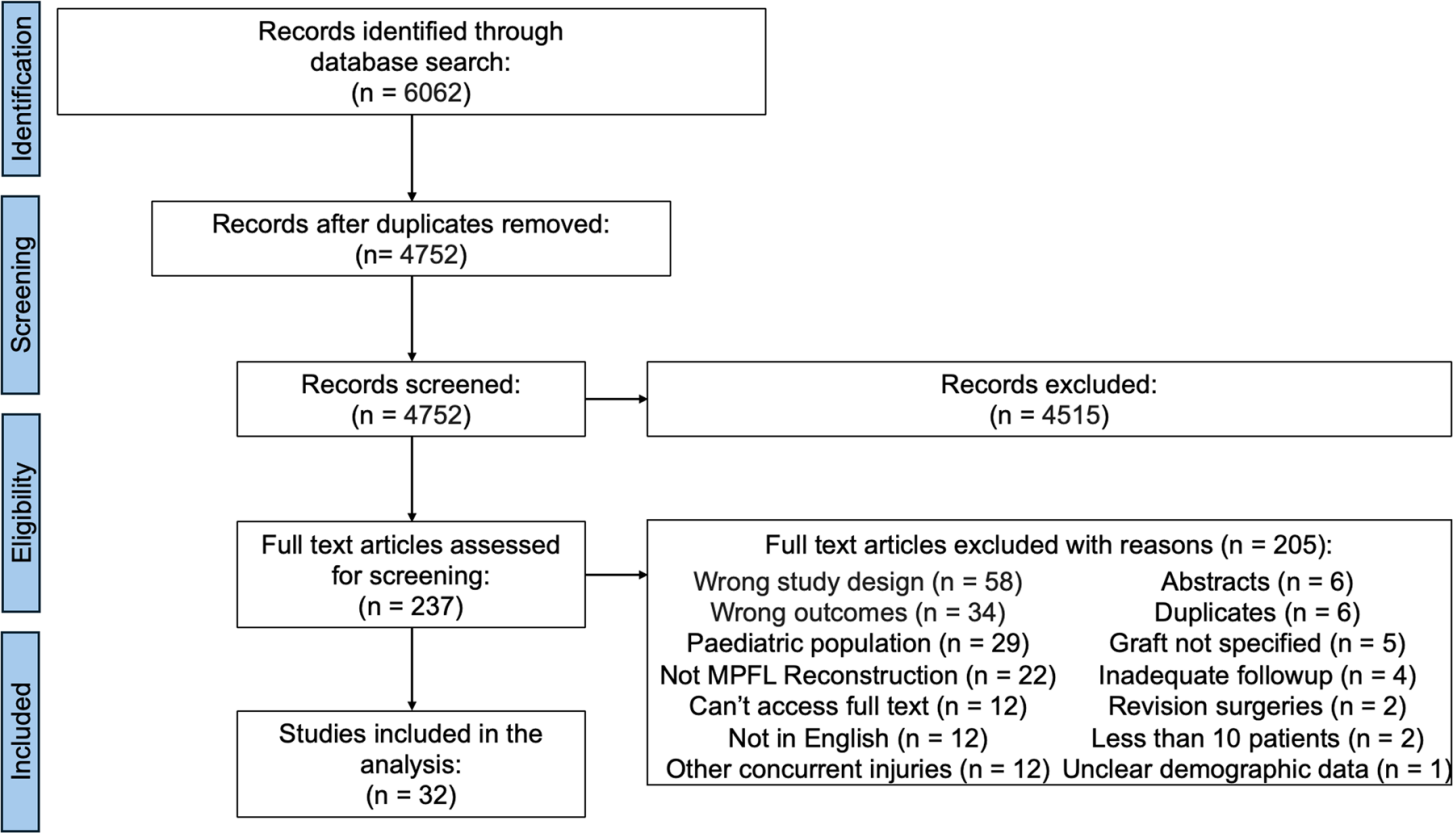
PRISMA. Study selection process showing records identified, screened, excluded and included in the final review and meta‐analysis. PRISMA, preferred reporting items for systematic reviews and meta‐analyses.

The search yielded 6062 records. After removing duplicates, 4752 records were screened, with 4515 excluded. Full texts of 237 articles were assessed, with 32 studies included [7, 10, 12, 14, 20, 22, 24, 26, 28, 29, 30, 31, 32, 34, 43, 44, 45, 46, 47, 49, 50, 51, 52, 55, 57, 60, 61, 64, 65, 66, 67, 68] (Figure 1). Of these, 4 investigated synthetic grafts, 27 biological grafts and 1 was comparative, encompassing a total of 1508 patients (1284 biological, 224 synthetic). In the studies that employed synthetic grafts, the pooled mean age was 24.0 years, the pooled body mass index (BMI) was 24.4 (*n* = 43), and 66.7% of patients were female. In the studies that employed biological grafts, the pooled mean age was 24.7 years, the pooled BMI was 24.6 (*n* = 523) and 61.8% of patients were female (*n *= 1274). The included studies were conducted across 14 countries, with the highest representation from China and the United Kingdom (*n* = 6 each), followed by Austria, France and Japan (*n* = 3 each). Study designs included prospective case series (*n* = 8), prospective cohort studies (*n* = 2) and randomised controlled trials (*n* = 4), with the remainder comprising retrospective cohort studies (*n* = 12), retrospective case series (*n* = 6) and one retrospective case–control study. Most studies were lower‐level evidence, consisting of 6 Level II (18.8%), 13 Level III (40.6%) and 13 Level IV (40.6%) studies. A full overview is provided in Tables 1 and 2.

**Table 1:** Study characteristics.

Title	Author	Study design (level of evidence)	Study location	*N*	Female (%)	Type of graft	Graft type	Category	Follow up (months)	Age	BMI	Conclusion
Evaluation of the results of reconstruction of the medial patellofemoral ligament in the treatment of recurrent patellar instability using Hamstring autograft by dual patella docking technique	Bayomy 2024	Prospective cohort (II)	Egypt	20	65.00	Hamstring tendon	Autograft	Biological	15.9	25.2		MPFL reconstruction with dual patella docking using hamstring autograft showed high success, improved Kujala and Lysholm scores and satisfactory joint congruence.
Medium‐term outcome of medial patellofemoral ligament reconstruction using synthetic graft	Deo 2023	Retrospective cohort (III)	UK	85	68.20	‐		Synthetic	53.76	28.06		Synthetic graft for MPFL reconstruction yields good outcomes and low complication rates, comparable to existing literature.
MPFL reconstruction using a quadriceps tendon graft: Part 2: Operative technique and short‐term clinical results	Fink 2014	Prospective case series (IV)	Austria	17	58.82	Quadricep tendon	Autograft	Biological	12	21.5		Minimally invasive QT graft technique shows good short‐term outcomes. A viable alternative for primary and revision MPFL in children and adults.
Clinical outcomes after medial patellofemoral ligament reconstruction: An analysis of changes in the patellofemoral joint alignment	Fujii 2021	Retrospective case series (IV)	Japan	27	77.78	Semitendinosus tendon	Autograft	Biological	12	23.9	21.9	MPFL‐R yields good outcomes but alignment may change within 3 months. Avoid lateral stress early post‐op.
Medial patellofemoral ligament (MPFL) reconstruction using quadriceps tendon autograft provides good clinical, functional and patient‐reported outcome measurements (PROM): a 2‐year prospective study.	Gföller 2019	Prospective case series (IV)	Austria	36	52.78	Quadricep tendon	Autograft	Biological	24	25.1	22	Quadriceps tendon graft MPFL‐R is safe and effective with good outcomes at 2 years.
Anatomical two‐bundle medial patellofemoral ligament reconstruction with hardware‐free patellar graft fixation: Technical note and preliminary results	Hinterwimmer 2013	Retrospective case series (IV)	Germany	19	68.00	Gracilis tendon	Autograft	Biological	16	23		
Medial patellofemoral ligament reconstruction: a prospective outcome assessment of a large single centre series.	Howells 2012	Prospective cohort study (IV)	UK	201	45.77	Semitendinosus tendon	Autograft	Biological	16	26		These findings add to existing evidence that MPFL reconstruction is an effective surgical procedure for selected patients with patellofemoral instability.
Medial patellofemoral complex reconstruction (combined reconstruction of medial patellofemoral ligament and medial quadriceps tendon‐femoral ligament) with semitendinosus autograft resulted in similar clinical and radiographic outcomes to medial patellofemoral ligament reconstruction in treating recurrent patellar dislocation	Hu 2024	Retrospective cohort (III)	China	72	68.06	Semitendinosus tendon	Autograft	Biological	52.1	22.3	23.5	MPFC‐R had similar outcomes to MPFL‐R. No added benefit in patients with Insall‐Salvati index > 1.2.
Minimally invasive medial patellofemoral ligament reconstruction for patellar instability using an artificial ligament: A 2‐year follow‐up	Khemka 2016	Retrospective case series (IV)	Australia	29	62.00	LARS 5 (16%) Merselene tape 17 (55%) Neoligament 9 (29%)		Synthetic	48	25		Minimally invasive synthetic MPFL‐R is safe with low redislocation risk and early mobilisation.
Modern synthetic material is a safe and effective alternative for medial patellofemoral ligament reconstruction.	Lee 2017	Prospective case series (IV)	UK	27	63.63	FibreTape		Synthetic	48	22		FiberTape MPFL‐R is safe and effective. It avoids tendon harvest complications with good outcomes. Both are techniques effective: double‐anchor showed better joint congruence and function.
				23	71.43	Gracilis tendon	Autograft	Biological	48	21		
Medial patellofemoral ligament reconstruction: A comparison of single‐bundle transpatellar tunnel and double‐anchor anatomic techniques for the treatment of recurrent lateral patellar dislocation in adults	Li 2019	RCT (II)	China	91	57.14	Semitendinosus tendon	Autograft	Biological	41.1	27.17		Compared with the single‐bundle transpatellar tunnel technique, the double‐anchor anatomic MPFLR technique may be more effective with a more congruous patellofemoral joint and better knee function. Both methods offer good results.
No difference in outcome between femoral soft‐tissue and screw graft fixation for reconstruction of the medial patellofemoral ligament: A randomised controlled trial	Lind 2019	RCT (II)	Denmark	60	63.00	Gracilis tendon	Autograft	Biological	24	24.65	24.75	Soft‐tissue fixation is equally effective and safe as screw fixation for MPFL‐R.
All aperture fixation technique of anatomical medial patellofemoral ligament (MPFL) reconstruction with semitendinosus double loop graft: A retrospective case series	Mahmoud 2021	Retrospective case series (IV)	Egypt	10		Semitendinosus tendon	Autograft	Biological	19.4	26		Effective, reproducible technique for restoring joint stability and function.
Lateral retinacular release is not recommended in association to MPFL reconstruction in recurrent patellar dislocation	Malatray 2019	RCT (II)	France	16	78.95	Gracilis tendon	Autograft	Biological	12	28	20.7*	No benefit of adding lateral release to MPFL‐R.
Isolated reconstruction of the medial patellofemoral ligament with an elastic femoral fixation leads to excellent clinical results	Marot 2021	Prospective cohort (II)	France	57	65.00	Gracilis Tendon	Autograft	Biological	>24	23.7		At 2–5 years’ follow‐up, isolated quasi‐anatomical MPFL reconstruction with a gracilis tendon autograft provides outcomes equivalent to isolated anatomical MPFL reconstruction in patients with recurrent patellar dislocation, including those without trochlear dysplasia and those with type A or B trochlear dysplasia.
Medial patellofemoral ligament reconstruction with a divergent patellar transverse 2‐tunnel technique.	Panni 2011	Case series (IV)	Italy	48	77.08	Semitendinosus tendon	Autograft	Biological	24	28		Modified MPFLR shows strong early/mid‐term outcomes with low redislocation and high satisfaction.
Medial patellofemoral ligament reconstruction using pedicled quadriceps tendon autograft yields similar clinical and patient‐reported outcomes but less donor‐site morbidity compared with Gracilis tendon autograft	Runer 2024	Retrospective case‐control (III)	Austria	64	46.88	Gracilis or quadricep tendon	Autograft	Biological	27.8	20	22.95	QT and GT MPFL‐R yielded similar outcomes; GT had more sensory issues.
Clinical outcomes of medial patellofemoral ligament reconstruction using FiberTape and knotless SwiveLock anchors	Sasaki 2022	Retrospective cohort (III)	Japan	43	68.89	FiberTape		Synthetic	24	19.4	24.4	FiberTape/SwiveLock technique safe with no redislocations, and improved KOOS scores at 2 years.
Clinical and radiological outcome of medial patellofemoral ligament reconstruction with a semitendinosus autograft for patella instability	Schottle 2005	Retrospective cohort (III)	Switzerland	12	66.67	Semitendinosus tendon	Autograft	Biological	47.5	30.1		MPFL reconstruction improves clinical symptoms, reduces the patellar tilt substantially, and may correct patella alta.
Functional outcomes after medial patellofemoral ligament reconstruction show an inverted J‐shaped relation with body mass index.	Sharma 2023	Retrospective cohort (III)	UK	97	50.50	Gracilis tendon	Autograft	Biological	12	24.6	28.4	BMI shows an inverted J‐shaped relationship with functional outcomes following MPFL reconstruction, without affecting complication or redislocation rates. In the absence of patella alta and severe trochlear dysplasia, isolated MPFL reconstruction is safe and effective, with optimal functional outcomes observed in patients with a BMI of approximately 20–21.
At 10‐year minimum follow‐up, one‐third of patients have patellofemoral arthritis after isolated medial patellofemoral ligament reconstruction using Gracilis tendon autograft	Shatrov 2023	Retrospective cohort (III)	France	50	68.50	Gracilis tendon	Autograft	Biological	76.8	25.4	23.2	Effective long‐term. 1/3 developed patellofemoral arthritis after 10 years.
Anatomic medial patellofemoral ligament reconstruction using patellar suture anchor fixation for recurrent patellar instability	Song 2014	Case series (IV)	Republic of Korea	20	50.00	Hamstring Tendon	Autograft	Biological	34.5	21*		Suture anchor MPFLR reliable with outcomes comparable to bone tunnel methods.
Medial patellofemoral ligament reconstruction in patients with lateral patellar instability and trochlear dysplasia	Steiner 2006	Case series (IV)	USA	34	65.00	Quadricep tendon autograft and patellar tendon allograft	Allograft/autograft	Biological	66.5	24		MPFLR provides durable pain relief, functional recovery, and effective prevention of recurrent dislocation in patients with patellar instability and femoral trochlear dysplasia, despite reduced bony constraint.
Reconstruction of the medial patellofemoral ligament using a synthetic graft with arthroscopic control of patellofemoral congruence	Suganuma 2016	Retrospective cohort (III)	Japan	40	66.67	Poly‐tape		Synthetic	24	20.7		Following MPFL reconstruction with a synthetic graft for recurrent patellar dislocation, slightly lateral patellar positioning relative to the trochlear centre was associated with better subjective outcomes than strict central reduction, without significant differences in objective knee function.
Clinical and radiological results after one hundred fifteen MPFL reconstructions with or without tibial tubercle transfer in patients with recurrent patellar dislocation‚ Äîa mean follow‐up of 5.4¬†years	Tscholl 2020	Retrospective cohort (III)	Switzerland	72	77.78	Gracilis tendon	Autograft	Biological	64.8	25.2		MPFLR with/without TTT reliable mid‐term; persistent pain not linked to anatomy.
An evaluation of the effectiveness of medial patellofemoral ligament reconstruction using an anatomical tunnel site	Valkering 2017	Case series (IV)	UK	31	67.74	Gracilis tendon	Autograft	Biological	37.2	23.9	27.8	Gracilis graft with anatomical tunnel placement produced good results; underscores need for thorough pre‐op planning.
Isolated reconstruction of the medial patellofemoral ligament with autologous quadriceps tendon.	Vavalle 2016	Retrospective cohort (III)	Italy	16	43.75	Quadricep tendon	Autograft	Biological	38	22		QT autograft MPFLR safe, effective, and avoids patella fracture. Suitable for skeletally immature patients.
Medial patellofemoral ligament reconstruction using a bone groove and a suture anchor at patellar: A safe and firm fixation technique and 3‐year follow‐up study.	Wang 2016	Retrospective cohort (III)	China	26	61.54	Gracilis tendon	Autograft	Biological	38.2	26.3		Combined bone groove and suture anchor fixation provides a safe, stable, and effective patellar fixation technique for MPFL reconstruction, with significant radiographic and functional improvements and no patellar complications at 3‐year follow‐up; safe drilling angles vary by Wiberg patellar type and sex, requiring progressively more vertical orientation from type I to III and a more oblique trajectory in female patients.
An isolated medial patellofemoral ligament reconstruction with patellar tendon autograft	Witonski 2013	Retrospective cohort (III)	Poland	10	60.00	Patellar tendon	Autograft	Biological	24	27.2		Medial 1/3 patellar tendon autograft MPFLR is simple, cost‐effective, and spares other grafts.
Medial patella‐femoral ligament reconstruction using the anterior half of the peroneus longus tendon as a combined procedure for recurrent patellar instability	Xu 2016	Case series (IV)	China	40	90.00	Anterior half of the peroneus longus tendon	Autograft	Biological	24	26.1		AHPLT is a promising graft with good static and functional outcomes when combined with adjunct procedures.
Comparison of clinical and radiological outcomes with body mass index after medial patellofemoral ligament reconstruction	Zhan 2024	Retrospective cohort (III)	China	70	64.30	Semitendinosus tendon	Autograft	Biological	26.73	22.52	24.43	Both BMI groups improved. Higher BMI had worse pre‐op function but similar radiologic gains.
The role of medial retinaculum plication versus medial patellofemoral ligament reconstruction in combined procedures for recurrent patellar instability in adults	Zhao 2012	RCT (II)	China	45	82.22	Semitendinosus tendon	Autograft	Biological	60	25		MPFLR yielded better static patella positioning and functional outcomes than plication.

*Note*: Summary of study design, level of evidence, location, inclusion criteria, sample size, sex distribution, graft type, follow‐up duration, age, BMI and study conclusions for patients undergoing MPFL reconstruction.

Abbreviations: ACL, anterior cruciate ligament; BMI, body mass index; CT, confidence interval; GT, gracilis tendon; LARS, ligament augmentation and reconstruction system; MPFL, medial patellofemoral ligament; MPFL‐R, medial patellofemoral ligament reconstruction; MPROP, mean percentage of the range of possible; MRAW, mean raw; MRI, magnetic resonance imaging; OA, osteoarthritis; PF, patellofemoral; QT, quadriceps tendon; TTT AHPLT, anterior hlaf of peroneus longus tendon; TT–TG, tibial tubercle–trochlear groove distance.

* Median.

### Results of risk of bias assessment

The RoB 2 tool was used to assess four randomised controlled trials, with three rated as low risk of bias and one judged to have some concerns due to missing outcome data. The ROBINS‐I tool was applied to 28 nonrandomised studies. Of these, 13 were rated as having moderate risk of bias, while the remaining 15 were rated as serious risk, primarily due to confounding and participant selection. Supporting Information: Tables S2 and S3 provide detailed domain‐level assessments for each study.

### Primary outcomes: Patient‐reported outcome measures

#### Kujala score

**Figure 2:**
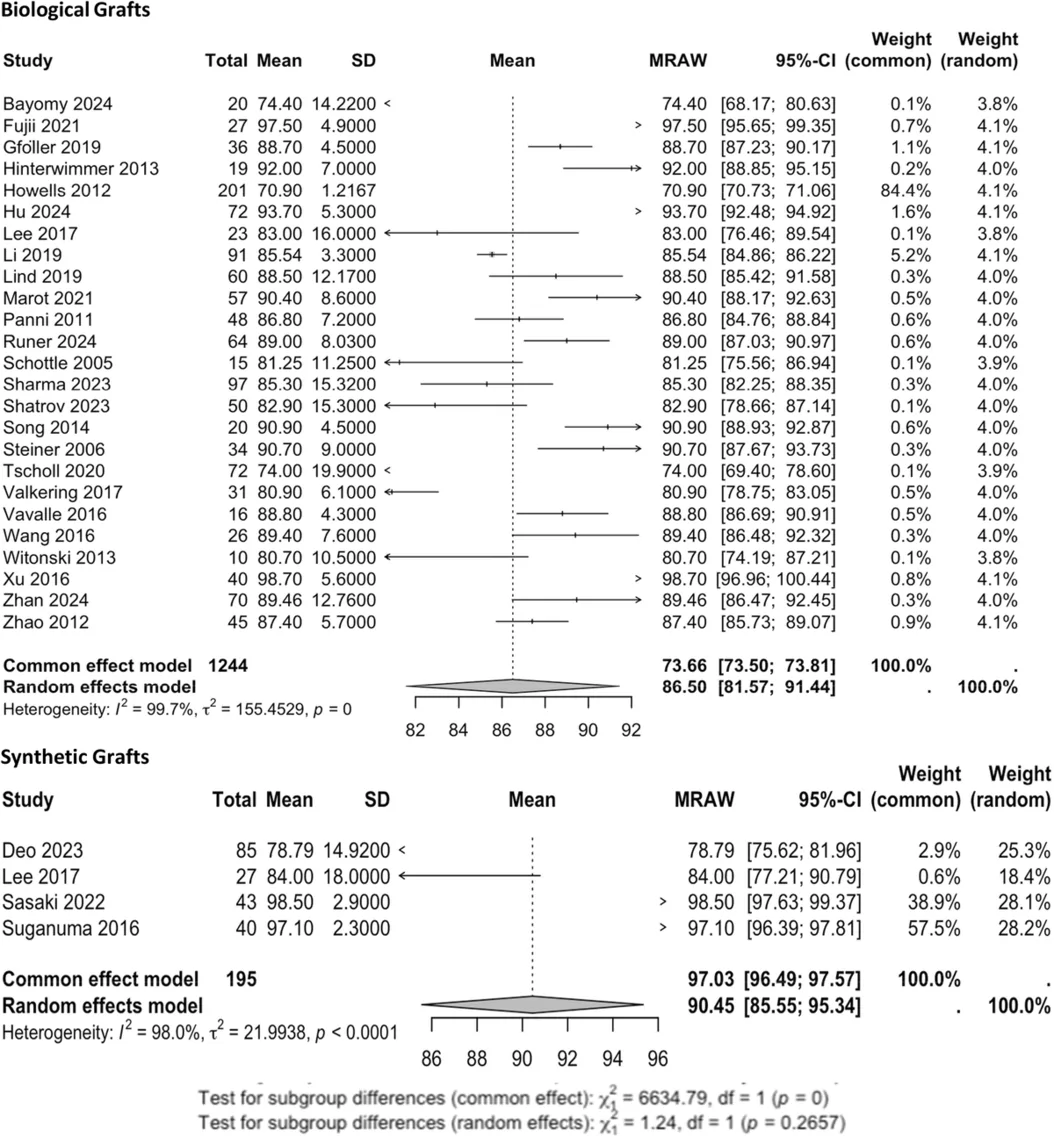
Kujala scores. Forest plots for pooled postoperative Kujala scores for included studies, following MPFL reconstruction. CI, confidence interval; MPFL, medial patellofemoral ligament; SD, standard deviation.

Across the 23 applicable studies utilising biological‐grafts (*n* = 1244), the pooled postoperative Kujala score was 86.50 (95% CI 81.57–91.44; τ² = 155.45; *I*² = 99.7%; *p* < 0.001) (Figure 2). In four studies using synthetic‐grafts (*n* = 195), the corresponding estimate was 90.45 (95% CI 85.55–95.34; τ² = 21.99; *I*² = 98.0%; *p* < 0.01). Although the synthetic subgroup trended higher, the *Q*‐test for subgroup differences was nonsignificant (*Q* = 1.24, df = 1, *p* = 0.27). Both subgroups displayed extreme heterogeneity.

#### Lysholm score

**Figure 3:**
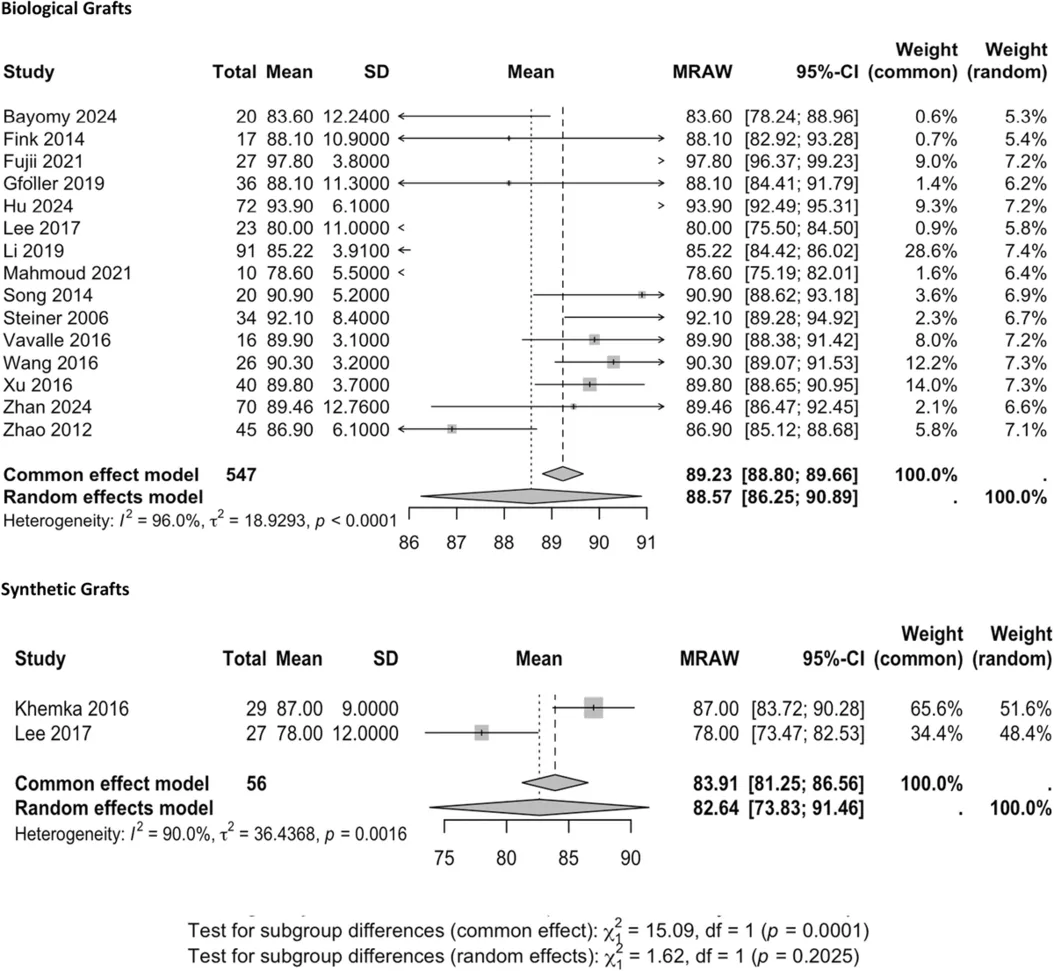
Lysholm scores. Forest plots for pooled postoperative Lysholm scores for included studies, following MPFL reconstruction. CI, confidence interval; MPFL, medial patellofemoral ligament; SD, standard deviation.

In 17 studies using biological grafts (*n* = 547), the pooled Lysholm score under a random‐effects model was 88.57 (95% CI 86.25–90.89; τ² = 18.93; *I*² = 96.0%; *p *< 0.001) (Figure 3). In the two studies that utilised synthetic grafts (*n* = 56), the corresponding summary was 82.64 (95% CI 73.83–91.46; τ² = 36.44; *I*² = 90.0%; *p* = 0.002). There was no evidence of a significant difference between groups (*Q*‐test for subgroup differences: *Q* = 1.62, df = 1, *p* = 0.20). Both subgroups exhibited considerable heterogeneity that remained unexplained.

#### Tegner score

**Figure 4:**
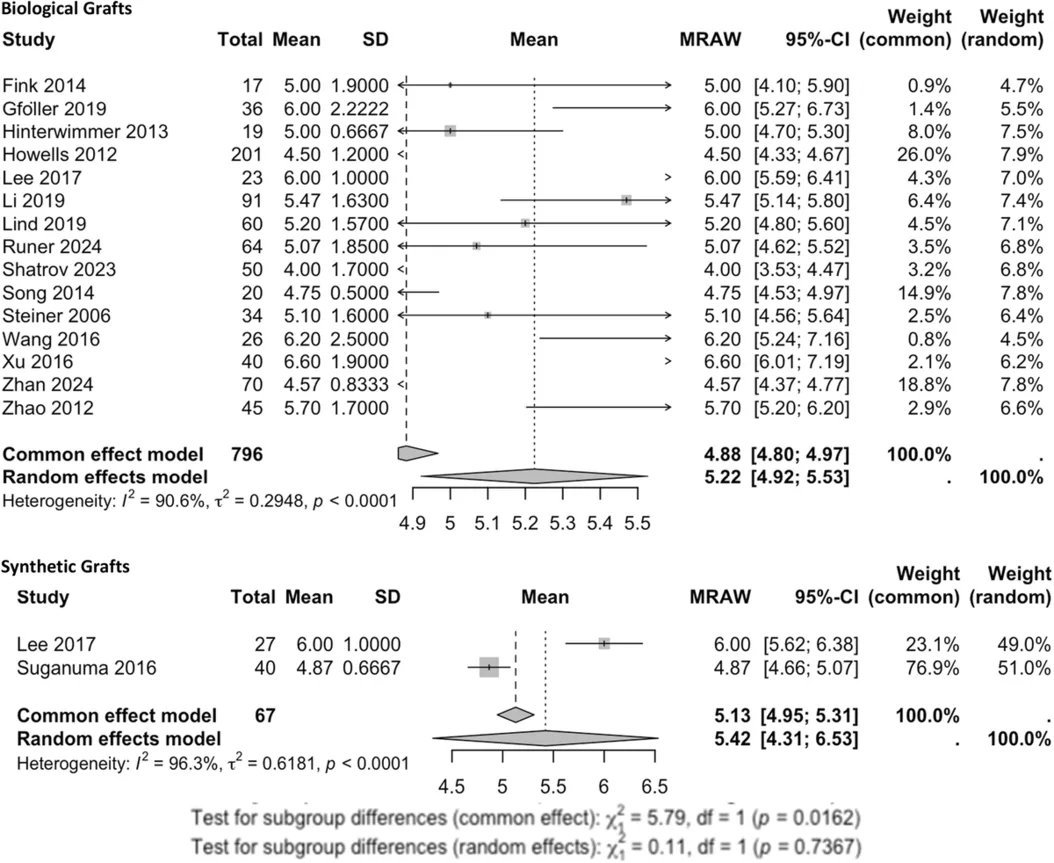
Tegner score. Forest plots for pooled postoperative Tegner scores for included studies, following MPFL reconstruction. CI, confidence interval; MPFL, medial patellofemoral ligament; SD, standard deviation.

Of the 15 applicable studies of biological grafts (*n* = 796), the pooled postoperative Tegner score under a random‐effects model was 5.22 (95% CI 4.92–5.53; τ² = 0.29; *I*² = 90.6%; *p* <0.001) (Figure 4). In the two studies of synthetic grafts (*n* = 67), the corresponding summary was 5.42 (95% CI 4.31–6.53; τ² = 0.62; *I*² = 96.3%; *p* < 0.001). Although synthetic grafts trended slightly higher, there was no evidence of a significant difference between groups (*Q*‐test for subgroup differences: *Q* = 0.11, df = 1, *p* = 0.74). Both subgroups exhibited substantial heterogeneity that remained unexplained.

#### IKDC score

Of the 10 applicable studies of biological grafts (*n* = 702), the pooled postoperative IKDC score under a random‐effects model was 78.94 (95% CI 75.48–82.41; τ² = 26.40; *I*² = 95.8%; *p* < 0.001) (Supporting Information: Figure S1). The synthetic‐graft subgroup had fewer than two studies and was therefore not analysed. However, Suganuma et al. reported a postoperative IKDC mean of 87.05 (SD: 11.1), suggesting potentially comparable outcomes. Both the magnitude and precision of the IKDC estimate for biological grafts should be interpreted in light of the very high residual heterogeneity.

### Secondary outcomes

#### Postoperative complication rate

**Figure 5:**
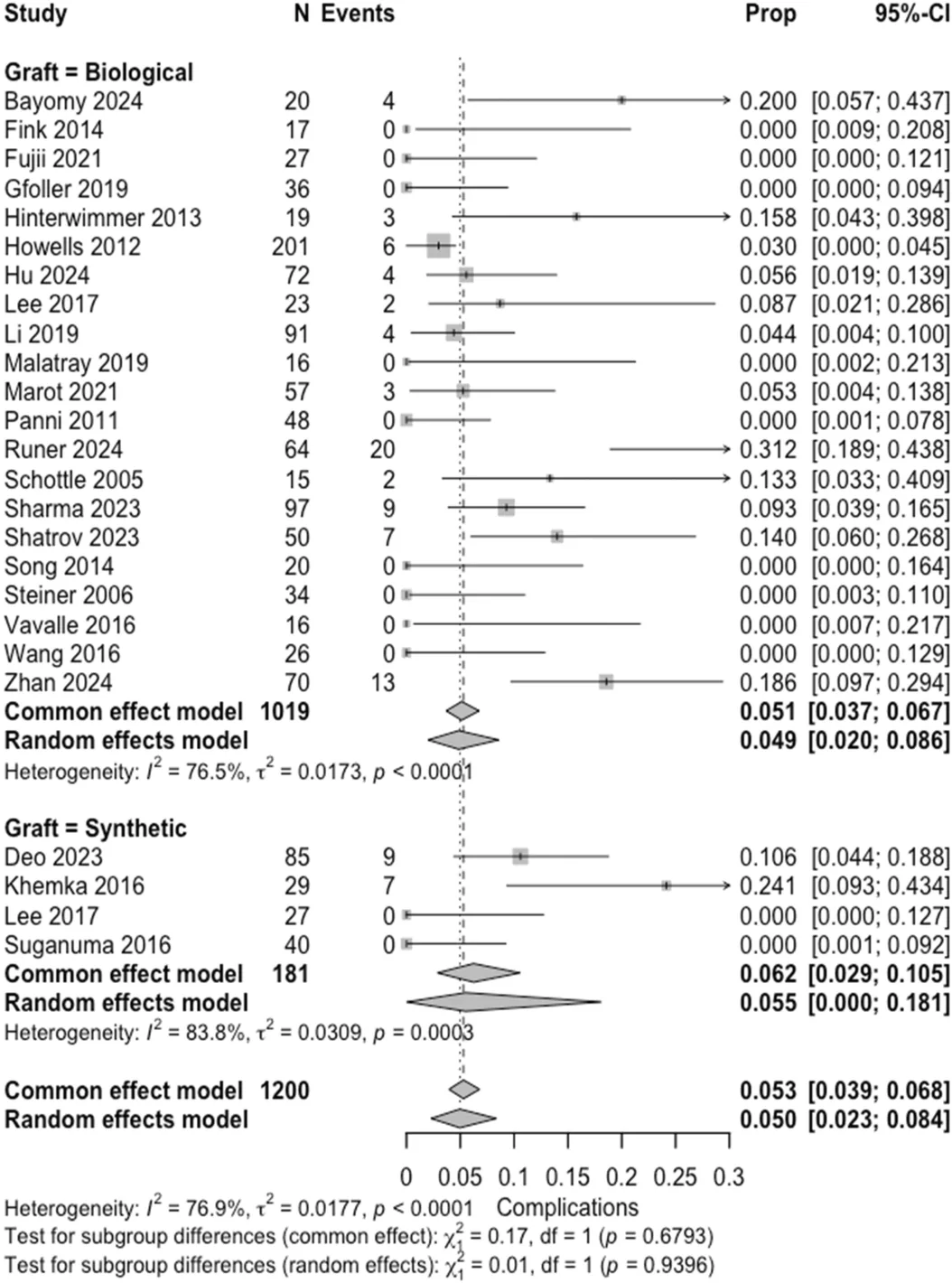
Postoperative complications. Forest plots for pooled postoperative complication rates for included studies, following MPFL reconstruction. CI, confidence interval; MPFL, medial patellofemoral ligament.

Of the 21 applicable studies of biological grafts (*n* = 1019), the pooled postoperative complication rate under a random‐effects model was 4.9% (95% CI 2.0%–8.5%; τ² = 0.02; *I*² = 76.5%; *p* < 0.001) (Figure 5). In the four studies of synthetic grafts (*n* = 181), the corresponding rate was 5.5% (95% CI 0.0%–18.1%; τ² = 0.03; *I*² = 83.8%; *p* < 0.001). Although synthetic grafts trended toward a higher complication rate, there was no significant difference between subgroups (*χ*² = 0.01, df = 1, *p* = 0.94). Both subgroups exhibited substantial heterogeneity. Postoperative pain and dislocation were the most common complications (Table 2).

**Table 2:** Outcomes of enroled patients in the included studies.

Author	Graft type	Kujala	Lysholm	Tegner	IKDC	Overall postoperative complication *n* (%)	Postoperative complication (%)	Positive patellar apprehension	Redislocation
Bayomy 2024	Biological	Baseline: 49.6 15.9 month: 74.4	Baseline: 57.8 15.9 month: 83.6		Baseline: 43.1 15.9 month: 68.4	4 (20)	Patellofemoral pain: 2 (10), residual subluxation: 1 (5), limited flexion: 1 (5)		
Deo 2023	Synthetic	Baseline: 42.12 53.76 month: 78.79				9 (10.6)	Medial knee pain: 5 (5.8), wound infection: 2 (2.4), residual instability: 2 (2.4)		0 (0)
Fink 2014	Biological		Baseline: 69.5 12 month: 88.1	Baseline: 4.8 12 month: 5		0 (0)	‐	12 month: 2 (11.8)	0 (0)
Fujii 2021	Biological	Baseline: 65.4 12 month: 97.5	Baseline: 79.4 12 month: 97.8			0 (0)	‐		0 (0)
Gföller 2019	Biological	Baseline: 82.1 12 month: 88.2 24 months: 88.7	Baseline: 79.3 12 month: 88.1 24 month: 90.2	Baseline: 6 12 month: 6 24 month: 6		0 (0)	‐		0 (0)
Hinterwimmer 2013	Biological	16 month: 92		16 month: 5*		3 (16)	Pain 2: (10.5), traumatic fracture: 1 (5.3)		0 (0)
Howells 2012	Biological	Baseline: 55.4 16 month: 81.69			Baseline: 45.98 16 month: 75.12	6 (3.3)	Superficial wound infection: 2 (1.1), deep vein thrombosis: 1 (0.6), neuroma at graft harvest site: 1 (0.6), mispositioned patellar tunnel requiring revision: 1 (0.6), traumatic fracture: 1 (0.6)		
Hu 2024	Biological	Baseline: 60.3 52.1 month: 93.7	Baseline: 66 52.1 month: 93.9	Baseline: 2.8 52.1 month: 4.5	Baseline: 55 52.1 month: 86	4 (5.56)	Instability: 4 (5.6)		
Khemka 2016	Synthetic		Baseline: 20 24 month: 87			7 (14.58)	Re‐dislocation: 1 (3.2), ligament prominence over medial femoral condyle: 4 (12.9), anterior knee pain at 24‐month follow‐up: 3 (9.7)		1 (3.2)
Lee 2017	Synthetic	Baseline: 64 12 month: 85 24 month: 82 48 month: 84	Baseline: 61 12 months: 81 24 month: 80 48 month: 78	Baseline: 3* 12 month: 6 24 Month: 6* 48 Month:6*		0 (0)	‐		0 (0)
Lee 2017	Biological	Baseline: 62 12 month: 86 24 month: 84 48 month: 83	Baseline: 59 12 month: 83 24 month: 80 48 month: 80	Baseline: 3* 12 month: 6* 24 month: 6* 48 Month: 6*		2 (4.16)	Superficial infections: 2 (4.16)		0 (0)
Li 2019	Biological	Baseline: 64.88 24 month: 85.54	Baseline: 53 41.1 months: 85.22	Baseline: 3.83 41.1 month: 5.47	Baseline: 48.99 41.1 month: 75.95	4 (4.4)	Instability: 4 (4.4)		0 (0)
Lind 2019	Biological	Baseline: 67.95 12 months: 82.5 24 month: 88.5		12 month: 5.25 24 month: 5.2			‐		0 (0)
Mahmoud 2021	Biological		Baseline: 59 19.4 month: 80.2				‐	47.5 month: 0 (0)	
Malatray 2019	Biological				Baseline: 54 12 month: 82	0 (0)	‐		
Marot 2021	Biological	Baseline: 61.58 > 24 month: 90.4				3 (5.4)	Instability 2 (3.6); redislocation: 1 (1.8)		1 (1.8)
Panni 2011	Biological	Baseline: 56.7 24 month: 86.8				0 (0)	‐		0 (0)
Runer 2024	Biological	24 month: 89	24 month: 87.1	24 month: 5.07		20 (31.25)	Sensory loss: 20 (31.25)		0 (0)
Sasaki 2022	Synthetic	12 month: 96.6 24 month: 98.5					‐	12 month = 2 (3.2) 24 month 1 (2.4)	0 (0)
Schottle 2005	Biological	Baseline: 53.3 47.5 month: 85				2 (13.3)	Instability: 2 (13.3)	19.4 months: 4 (25.7)	1 (6.7)
Sharma 2023	Biological	Baseline: 62.9 12 month: 85.3			Baseline: 50 12 month: 74.4	9 (9.28)	Chondroplasty for mechanical symptoms: 3 (5.3), Painful hardware removal: 2 (3.5), Manipulation under anaesthesia (MUA) for arthrofibrosis: 1 (1.8), Excision of scar: 1 (1.8), MUA for arthrofibrosis: 2 (5.6)		0 (0)
Shatrov 2023	Biological	76.8 month: 82.9		76.8 month: 4	76.8 month: 78.4	7 (14)	Recurrent patellar dislocation requiring revision surgery: 4 (7.4), postoperative haematoma: 1 (1.9), ACL reconstruction: 1 (1.9), arthroscopic arthrolysis: 1 (1.9).		4 (7.4)
Song 2014	Biological	Baseline: 52.6 34.5 month: 90.9	Baseline: 49.2 34.5 months: 90.9	Baseline: 3* 34.5 month: 5*		0 (0)	‐	34.5 month: 1 (5)	0 (0)
Steiner 2006	Biological	Baseline: 53.3 66.5 month: 90.7	Baseline: 52.4 66.5 month: 92.1	Baseline: 3.1 66.5 month: 5.1		0 (0)	‐		0 (0)
Suganuma 2016	Synthetic	Baseline: 74.2 24 month: 97.1		Baseline: 4.6 24 month: 4.6	Baseline: 69.6 24 month: 87.05	0 (0)	‐		0 (0)
Tscholl 2020	Biological	Baseline: 52.7 64.8 month: 74					(Omitted due to follow‐up being significantly longer than other studies, skewing outcomes		3 (3.4)
Valkering 2017	Biological	Baseline: 53.3 37.2 month: 80.9					‐		
Vavalle 2016	Biological	Baseline: 35.8 38 month: 88.8	Baseline: 43.3 38 months: 89.9			0 (0)	‐		0 (0)
Wang 2016	Biological	Baseline: 53.2 38.2 month: 89.4	Baseline: 59.6 38.2 months: 90.3	Baseline: 3.1 38.2 month: 6.2		0 (0)	‐		0 (0)
Witonski 2013	Biological	Baseline: 59.7 24 month: 84.4					‐		0 (0)
Xu 2016	Biological	Baseline: 73.8 12 month: 94.4 24 month: 98.7	Baseline: 58 12 months: 89.8 24 month: 94.3	Baseline: 3.6 12 month: 6.1 24 month: 6.6	Baseline: 52.1 12 month: 81.9 24 month: 85.1		‐	Baseline = 28 (70) 12 month = 1 (2.5) 24 month 1 (2.5)	0 (0)
Zhan 2024	Biological	Baseline: 52.53 26.73 month: 89.46	Baseline: 58.38 26.73 month: 89.46	Baseline: 1.57 26.73 month: 4.71	Baseline: 49.51 26.73 month: 80.61	13 (18.6)	‐		0 (0)
Zhao 2012	Biological	Baseline: 68.9 24 month: 86.3 60 month: 87.4	Baseline: 52.1 24 month: 8560 month: 86.9	Baseline: 3.1 24 month: 5.4 60 month: 5.7	Baseline: 46.3 24 month: 75.9 60 month: 79.4		‐		1 (6.3)

*Note*: Reported postoperative outcome scores (Kujala, Lysholm, Tegner and IKDC), complication rates, patellar apprehension and redislocation events across included studies. Where available, data are presented at baseline and at final follow‐up. Complication data are summarised as the number (%) of events.

Abbreviation: IKDC, International Knee Documentation Committee.

* Median.

#### Redislocation rates

**Figure 6:**
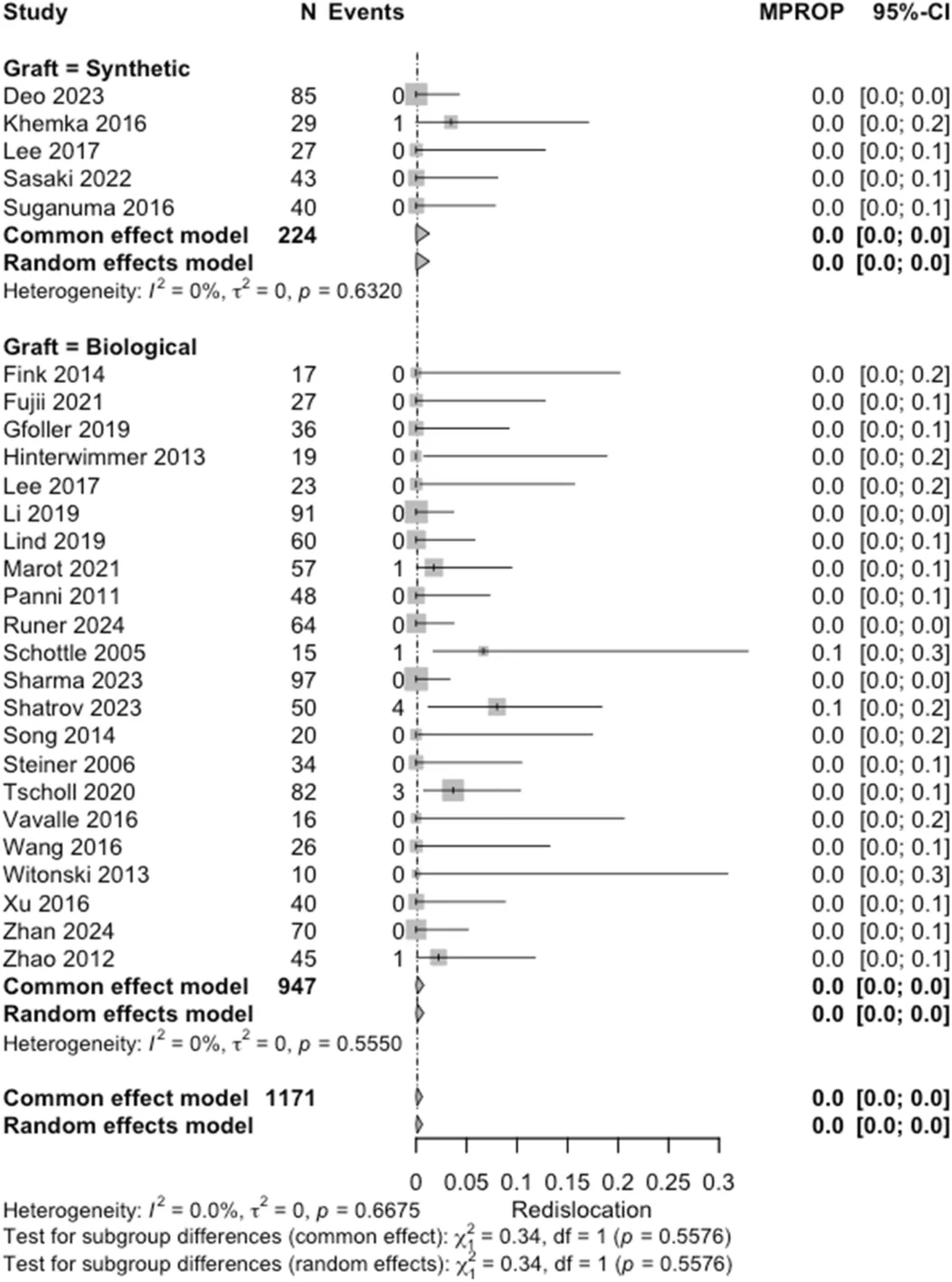
Redislocation rates. Forest plots for pooled redislocation rates for included studies, following MPFL reconstruction. CI, confidence interval; MPFL, medial patellofemoral ligament.

Of the 22 applicable studies of biological grafts (*n *= 1171), the pooled postoperative complication rate under a common‐effects model was 0.1% (95% CI 0.0%–0.7%; τ² = <0.; *I*² = 0%; *p* = 0.5550) (Figure 6). In the five studies of synthetic grafts (*n* = 224), the pooled rate was 0% (95% CI 0%–1.3%; τ² = 0; *I*² = 0%; *p* < 0.6320). There was no significant difference in redislocation rates between the graft types (*Q*‐test for subgroup differences: *Q* = 0.34, df = 1, *p* = 0.5576).

#### Patients with positive patellar apprehension test

In five studies of biological grafts (*n* = 102), the pooled postoperative patellar apprehension proportion under a random‐effects model was 7.0% (95% CI 1.0%–16.0%; τ² = 0.01; *I*² = 46.6%; *p* = 0.11) (Supporting Information: Figure S1). The synthetic‐graft subgroup included fewer than two studies and was therefore not meta‐analysed. The moderate heterogeneity among the biological‐graft studies (*I*² = 46.6%) was not statistically significant. The synthetic‐graft subgroup had fewer than two studies and was therefore not analysed. Sasaki et al. of the synthetic group had one patient (2.4%) with a positive test postoperatively, yielding potentially similar results.

#### Publication bias

Egger′s regression in the biological graft group showed significant small‐study effects for Kujala (*t* = 5.67, *p* < 0.01) and Tegner (*t* = 3.34, *p* < 0.01) postoperative scores, but no asymmetry for Lysholm (*t* = –0.03, *p* = 0.98) or IKDC (*t* = 0.73, *p* = 0.49) scores. Funnel plots can be found in Supporting Information: Figure S3. Analysis of publication bias was not performed for the synthetic group due to small sample sizes.

## DISCUSSION

The findings of this study indicate that there were no statistically significant differences between biological autografts or allografts and synthetic grafts in terms of Kujala score, Lysholm score, Tegner score, re‐dislocation rate or postoperative complication rate in adult patients (≥18 years) with a minimum follow‐up of 12 months. These findings suggest that synthetic grafts may be an acceptable option for MPFL reconstruction, producing equivocal clinical outcomes to biological grafts. This study is the first meta‐analysis to compare the functional outcomes between biological and synthetic grafts for MPFL reconstruction.

Autografts have been proposed as a preferable option to allografts for MPFL reconstruction, predominantly due to the potential risk of histocompatibility issues associated with the latter, despite comparable outcomes [3, 18, 38]. Autografts have demonstrated good clinical outcomes, as they are derived from the patient′s own tissue and are less likely to trigger an immune response or cause rejection [38, 39]. Conversely, utilising allografts may have advantages in preserving native tissue, as well as a lower risk of recurrent instability [21, 37, 38]. Furthermore, several studies have identified allografts as having a reduced risk of strength loss, quicker recovery times, shorter surgical procedures and suitability for patients with connective tissue disorders [6, 16, 40]. When assessing biomechanical comparability to a native MPFL, several studies have shown quadriceps tendon to have similar properties compared to the threefold tensile strength of hamstring tendons [9, 12, 58]. In contrast, newer synthetic grafts with a complete transverse knitted structure have shown similar tensile strength to native MPFL tissue, which may support their use as an adjunct [8]. Based on current evidence, the incidence of recurrent patellar dislocation appears to be low following MPFL reconstruction, regardless of whether an allograft or autograft tissue is used. The risk of recurrent instability appears to be similar for both types of grafts. As per Xu et al. and Flanigan et al., the dislocation risk can be further mitigated by supplementing a reconstructed MPFL with a tibial tubercle osteotomy (TTO) [13, 66]. However, not all cases of patellofemoral instability require a concomitant TTO, and the decision should be based on individual anatomical factors such as patellar height, tibial tuberosity–trochlear groove (TT–TG) distance and trochlear morphology.

The primary outcome measures of interest were patient‐reported functional outcomes scores. These included Kujala scores, Lysholm score, Tegner score and IKDC scores. Previous studies have shown no significant effect of graft type on Kujala score improvement, with all grafts demonstrating comparable enhancement [35, 59]. The findings of the present analysis are consistent with this, showing similar postoperative improvement in Kujala scores between biological and synthetic grafts. Likewise, in line with Ulrich et al., who reported significant visual analogue scale (VAS) improvements across graft types, our analysis found no significant differences between biological and synthetic grafts in VAS or other functional scores [59]. Similarly, no significant differences were observed between the biological and synthetic graft groups in terms of Lysholm, Tegner and IKDC scores, as well as re‐dislocation rates and patellar‐apprehension test findings. Taken together, these results indicate that synthetic grafts provide clinical outcomes comparable to biological grafts in MPFL reconstruction, while also avoiding drawbacks associated with biological grafts such as donor‐site morbidity, tendon harvesting, soft‐tissue irritation and postoperative discomfort [28].

However, the utilisation of synthetic ligaments in ligament reconstruction is limited by several factors, particularly with regards to their safety profile. Long‐term complications of synthetic ligaments include osteolysis, chronic effusions, synovitis and rupture, among others. Ventura et al.'s case series with 126 patients who underwent polyethylene ligament Anterior Cruciate Ligament reconstruction with a follow‐up period of 19 years, demonstrated a range of complications, including osteoarthritis (100%), re‐ruptures (27.5%) and functional impairment (29.4%) [62]. While such studies highlight the potential long‐term risks of synthetic materials in ligament reconstruction, there are very few equivalent studies evaluating synthetic grafts specifically within MPFL reconstruction, and long‐term outcomes in this context remain largely unknown. Technical considerations such as graft tensioning and the potential for foreign body reactions are also important when comparing biological and synthetic grafts. However, few studies provided sufficient detail to allow meaningful comparison of these factors. These technical aspects likely influence both graft performance and long‐term outcomes, and they represent important areas for further investigation. The findings of this review highlight the need for continued evaluation of both the clinical and economic implications of synthetic graft use as a treatment option for MPFL reconstruction. Future studies must also assess the cost‐effectiveness of synthetic grafts, as their higher material costs may not justify their use given the similar outcomes to biological grafts. Furthermore, allografts themselves are typically more expensive than autografts and may be subject to availability and regulatory constraints, which can further influence graft selection. However, when factoring in reoperation costs, allograft procedures have been shown to be less expensive overall than autograft surgeries as reported by Hendawi et al., although this study was conducted in a paediatric population and the findings may not fully reflect cost patterns in adults given the limited data currently available [3, 17]. The economic burden associated with synthetic ligament implants may also limit their widespread adoption, particularly in healthcare systems where cost containment and value‐based care are prioritised.

This review has some limitations that require attention. Most included studies were observational, with limited direct comparisons between synthetic and biological grafts, a moderate level of evidence and heterogeneity across study designs may have affected pooled outcomes. Functional outcomes were assessed using different scoring systems, introducing potential measurement bias. Follow‐up beyond 2 years was uncommon, so results should be interpreted as short‐ to mid‐term. Reporting of postoperative complications and redislocation was inconsistent, likely contributing to underreporting and reduced comparability. Anatomical exclusion criteria varied, and some studies included patients with factors such as patella alta, trochlear dysplasia or elevated TT–TG distance, potentially influencing outcomes. Restricting the included patient population to adults improved cohort homogeneity but excluded mixed or paediatric studies, reducing the number of eligible studies, particularly those evaluating synthetic grafts. Autografts and allografts could not be analysed separately because most studies reported only autograft outcomes and very few evaluated allografts or directly compared them with synthetic grafts, limiting the feasibility of graft‐specific subgroup analyses. To address these limitations, future work would benefit from standardised reporting tools for MPFL reconstruction outcomes and from prospective comparative studies with larger sample sizes, more consistent methodology and longer‐term follow‐up.

## CONCLUSION

This study revealed that there is no significant difference between biological and synthetic grafts in terms of Kujala score, Lysholm score, Tegner score, IKDC score, VAS, re‐dislocation rate and the number of patients with positive patellar apprehension test. These results suggest that synthetic grafts may be a suitable alternative to biological grafts and should be considered in clinical practice given the increasing number of patients undergoing MPFL reconstruction for patellofemoral instability. However, it is important to acknowledge that the included studies were limited to adult patients with a minimum follow‐up of 12 months, generally reported short‐ to mid‐term outcomes and were predominantly of lower evidence level, which should be considered when interpreting these findings. To validate these findings and determine the long‐term efficacy and safety of synthetic grafts, further large‐scale randomised controlled trials with longer follow‐up are warranted. Such studies would provide higher levels of evidence to analyse the use of synthetic grafts in MPFL reconstruction.

## AUTHOR CONTRIBUTIONS

**Saran Singh Gill**: Conceptualisation; methodology; formal analysis; investigation; writing—original draft; writing—review and editing. **Pratik Ramkumar**: Methodology; formal analysis; investigation; writing—original draft; writing—review and editing. **Akash Patel**: Supervision; writing—review and editing. **Fahad Siddique Hossain**: Supervision; writing—review and editing. **Raj R. Thakrar**: Conceptualisation; supervision; writing—review and editing.

## CONFLICT OF INTEREST STATEMENT

The authors declare no conflicts of interest.

## ETHICS STATEMENT

The authors have nothing to report.

## Supporting information

MPFL Supp material.

## Data Availability

The data that support the findings of this study are available from the corresponding author upon reasonable request.
