# Supplementary material for: Comparable clinical and functional outcomes between biological and synthetic grafts for medial patellofemoral ligament reconstruction: A systematic review and meta‐analysis
Source: J Exp Orthop. 2026 Feb 19;13(1):e70627. doi: 10.1002/jeo2.70627 (PMC12919371; doi:10.1002/jeo2.70627)
Supplement: Supplementary file 1 — MPFL Supp material. [file JEO2-13-e70627-s001.docx]

Supplementary material


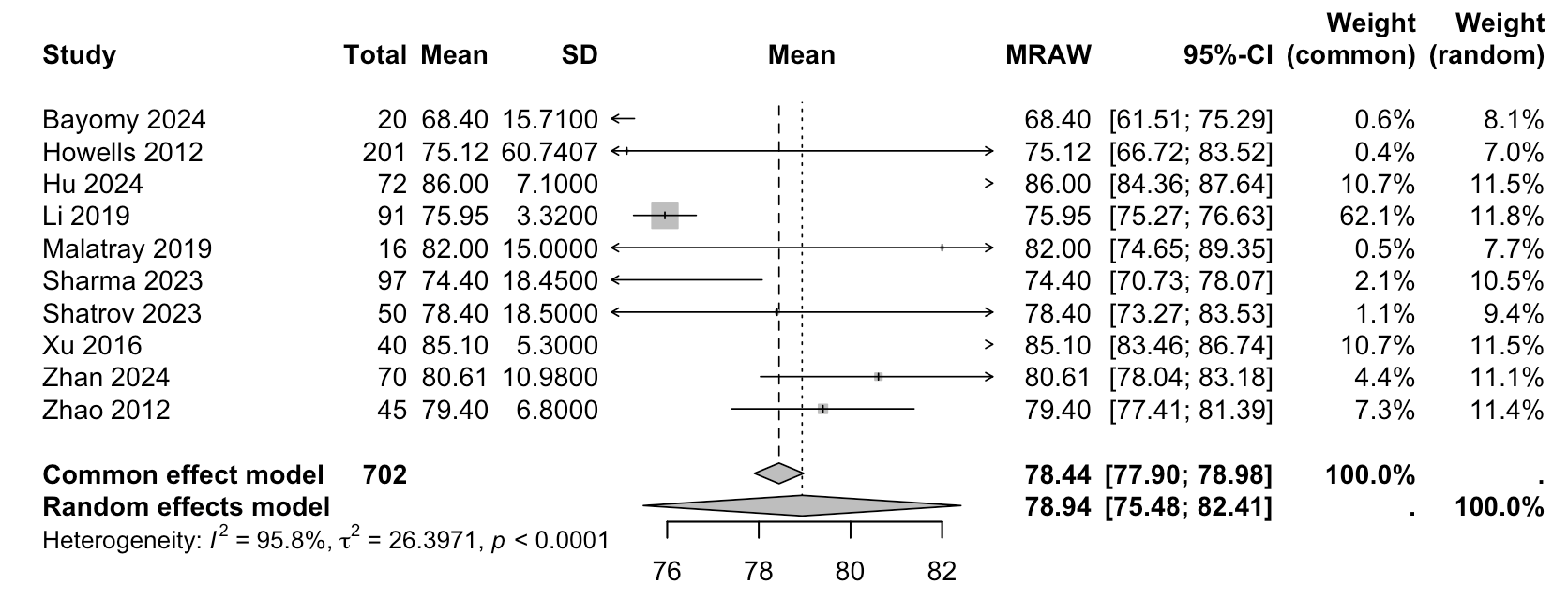


Figure S1: IKDC in Biological Grafts

Forest plots for pooled postoperative IKDC scores for included studies, following MPFL reconstruction.


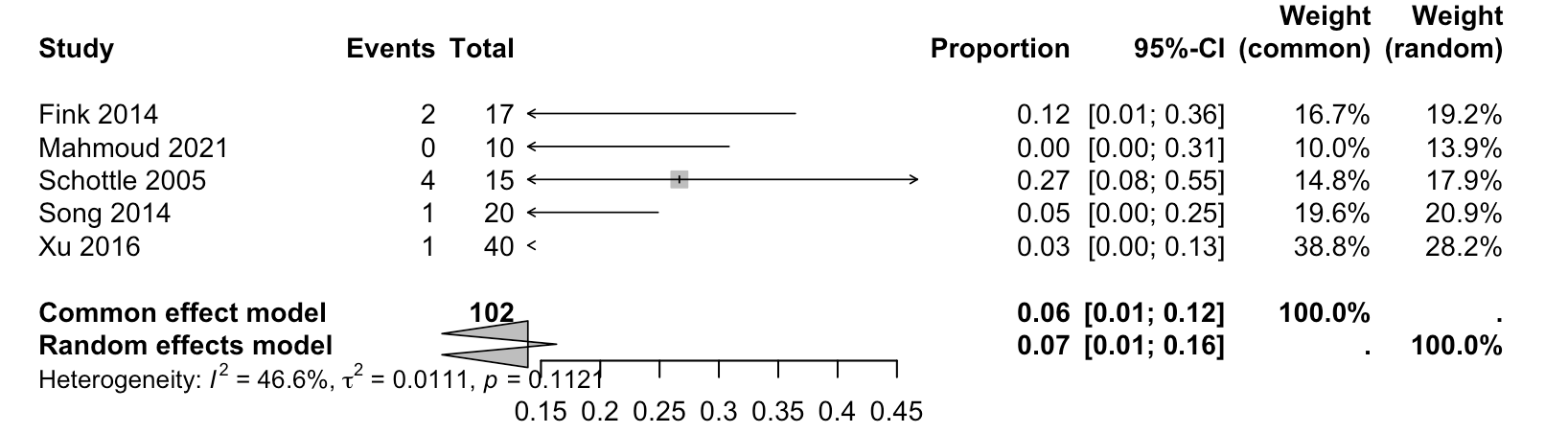


Figure S2: Patellar Apprehension in Biological Grafts

Forest plots for pooled rates of positive patellar apprehension tests in scores for included studies, following MPFL reconstruction.

Figure S3: Funnel Plots

Visual inspection of potential publication bias across included studies for the primary outcomes.

| Table S1, Search Strategy | | | |
| --- | --- | --- | --- |
| 1. | patell*[tiab] | 21. | graft[tiab] |
| 2. | “patellar dislocation” [tiab] | 22. | PROMS [tiab] |
| 3. | dislocation[tiab] | 23. | function*[tiab] |
| 4. | “patella dislocation” [tiab] | 24. | movement[tiab] |
| 5. | patellofemoral[tiab] | 25. | motion[tiab] |
| 6. | FiberTape[tiab] | 26. | flexion[tiab] |
| 7. | synthetic[tiab] | 27. | QOL [tiab] |
| 8. | “synthetic graft” | 28. | “quality of life” [tiab] |
| 9. | “autograft” [tiab] | 29. | safe[tiab] |
| 10. | Semitendinosus[tiab] | 30. | “range of motion” [tiab] |
| 11. | Gracilis[tiab] | 31. | dislocation[tiab] |
| 12. | Quadricep[tiab] | 32. | effective[tiab] |
| 13. | Quad[tiab] | 33. | EQ5D[tiab] |
| 14. | tendon[tiab] | 34. | kujala[tiab] |
| 15. | GT[tiab] |  |  |
| 16. | muscl*[tiab] |  |  |
| 17. | hamstring[tiab] |  |  |
| 18. | MPFL [tiab] |  |  |
| 19. | “medial patellofemoral ligament reconstruction” [tiab] |  |  |
| 20. | “medial patellofemoral” [tiab] |  |  |

/ = *Mesh term* ti = *Title* ab = *Abstract* * = *Truncation*MEDLINE search terms used for systematic identification of studies evaluating MPFL reconstruction, including truncations, title/abstract limits, and MeSH terms.

Table S2, Risk of Bias: RoB 2

| Study ID | Randomization Process (D1) | Deviations from Intended Intervention (D2) | Missing Outcome Data (D3) | Outcome Measurement (D4) | Selective Reporting (D5) | Overall RoB 2 Rating |
| --- | --- | --- | --- | --- | --- | --- |
| Li 2019 | Low risk | Low risk | Low risk | Low risk | Low risk | Good |
| Lind 2019 | Low risk | Low risk | Low risk | Low risk | Low risk | Good |
| Malatray 2019 | Low risk | Low risk | Some concerns | Low risk | Low risk | Some concerns |
| Zhao 2012 | Low risk | Low risk | Low risk | Low risk | Low risk | Good |

Assessment domains include randomization process, deviations from intended interventions, missing outcome data, outcome measurement, and selective reporting. Overall risk of bias rating is provided for each RCT.

Table S3, Risk of Bias: ROBINS-I

| Study ID | Confounding (D1) | Selection of Participants (D2) | Classification of Interventions (D3) | Deviations from Intended Intervention (D4) | Missing Data (D5) | Outcome Measurement (D6) | Selective Reporting (D7) | Overall ROBINS-I Rating |
| --- | --- | --- | --- | --- | --- | --- | --- | --- |
| Bayomy 2024 | Moderate | Moderate | Low risk | Low risk | Low risk | Low risk | Low risk | Moderate |
| Deo 2023 | Moderate | Moderate | Low risk | Low risk | Low risk | Low risk | Low risk | Moderate |
| Fink 2014 | Serious | Moderate | Low risk | Low risk | Low risk | Moderate | Low risk | Serious |
| Fujii 2021 | Serious | Moderate | Low risk | Low risk | Low risk | Low risk | Low risk | Serious |
| Gföller 2019 | Moderate | Moderate | Low risk | Low risk | Low risk | Low risk | Low risk | Moderate |
| Hinterwimmer 2013 | Serious | Moderate | Low risk | Low risk | Low risk | Low risk | Low risk | Serious |
| Howells 2012 | Serious | Low risk | Low risk | Low risk | Low risk | Low risk | Low risk | Serious |
| Hu 2024 | Moderate | Moderate | Low risk | Low risk | Low risk | Low risk | Low risk | Moderate |
| Khemka 2016 | Moderate | Moderate | Low risk | Low risk | Low risk | Low risk | Low risk | Moderate |
| Lee 2017 | Moderate | Low risk | Low risk | Low risk | Low risk | Low risk | Low risk | Moderate |
| Mahmoud 2021 | Moderate | Moderate | Low risk | Low risk | Low risk | Low risk | Low risk | Moderate |
| Marot 2021 | Moderate | Moderate | Low risk | Low risk | Low risk | Low risk | Low risk | Moderate |
| Panni 2011 | Serious | Serious | Low risk | Low risk | Low risk | Low risk | Moderate | Serious |
| Runer 2024 | Moderate | Moderate | Low risk | Low risk | Low risk | Low risk | Low risk | Moderate |
| Sasaki 2022 | Serious | Moderate | Low risk | Low risk | Low risk | Low risk | Low risk | Serious |
| Schöttle 2005 | Serious | Moderate | Low risk | Low risk | Low risk | Low risk | Low risk | Serious |
| Sharma 2023 | Moderate | Moderate | Low risk | Low risk | Low risk | Low risk | Low risk | Moderate |
| Shatrov 2023 | Serious | Moderate | Low risk | Low risk | Low risk | Low risk | Low risk | Serious |
| Song 2014 | Serious | Serious | Low risk | Low risk | Low risk | Low risk | Low risk | Serious |
| Steiner 2006 | Serious | Serious | Low risk | Low risk | Moderate | Low risk | Low risk | Serious |
| Suganuma 2016 | Moderate | Moderate | Low risk | Low risk | Low risk | Low risk | Low risk | Moderate |
| Tscholl 2020 | Moderate | Moderate | Low risk | Low risk | Low risk | Low risk | Low risk | Moderate |
| Valkering 2017 | Serious | Serious | Low risk | Low risk | Low risk | Low risk | Low risk | Serious |
| Vavalle 2016 | Serious | Serious | Low risk | Low risk | Low risk | Low risk | Low risk | Serious |
| Wang 2016 | Serious | Serious | Low risk | Low risk | Low risk | Low risk | Low risk | Serious |
| Witoński 2013 | Serious | Serious | Low risk | Low risk | Moderate | Low risk | Low risk | Serious |
| Xu 2016 | Moderate | Moderate | Low risk | Low risk | Moderate | Low risk | Low risk | Moderate |
| Zhan 2024 | Moderate | Moderate | Low risk | Low risk | Low risk | Low risk | Low risk | Moderate |

Risk of bias domains include confounding, selection of participants, classification of interventions, deviations from intended interventions, missing data, outcome measurement, and selective reporting. Overall ROBINS-I rating is provided.
